# New Drugs and Preparations

**Published:** 1894-04-28

**Authors:** 


### New Drugs and Preparations.

[We shall be glad to receive, at our Office, 428, Strand, London, W.C., from the manufacturers, specimens of all new preparations and drugs which may be brought out from time to time.]

### A PHARMACOLOGICAL STUDY OF AMYLENE HYDRATE.

Harnack and Meyer have made a more thorough pharmacological investigation[1] of this substance than has hitherto been done. It is a liquid, isomeric with amylic alcohol, and was recommended as a valuable hypnotic by V. Mering in 1887[2]. Since then several observers, chiefly in Germany, have confirmed his results, and have abundantly shown that in a half to one and a half drachm doses it causes satisfactory sleep lasting several hours, and closely resembling normal slumber. It acts more quickly and intensely than sulponal or paraldehyde, and is much less depressing to the circulation than chloral hydrate. Untoward effects, such as nausea and heaviness on waking, have been of only very occasional occurence, and even after extreme doses in man nothing more hurtful than intoxication has been noted. Jeskow[3] and Vivante[4] found, however, that it greatly lowered the temperature, and was capable of doing so even to an alarming degree. This is confirmed by Harnack and Meyer, who find that in rabbits non-lethal doses will lower the temperature as much as 12 deg. Fah., while fatal doses may do so 21 deg. Fah. In dogs, however, an ordinary hypnotic dose had no apparent effect on the temperature, although larger amounts might diminish it from 5 deg. to 10 deg. Fah. In dogs, sleep was preceded by a short period of excitement, and the authors are of opinion that the substance acts essentially like ethylic alcohol, but is much more active and concentrated. The respiration and blood-pressure were both depressed in animals, and observation on one of the authors showed that after one-half to one drachm doses there was a distinct fall of temperature with slowing, softness, and some dicrotism of the pulse. It is antagonistic to convulsive poisons like strychnine, santonin, and picrotoxin. In passing the author's note that santonin has an antipyretic action, and that a combination of it with amylene hydrate is strongly antipyretic. It diminishes the output of urea and greatly lessens general metabolism. Besides its hypnotic action it has been used with some success in epilepsy[5] and in chorea, and the authors suggest that trial should be made of its antipyretic value, either alone or combined with santonin. Peiser[6] finds that amylene hydrate diminishes the excretion of urea by about thirty grains in the twenty-four hours, and therefore as it lessens metabolism, he concludes that it would be a most suitable hypnotic to use in fever, diabetes, phthisis, and other wasting diseases, where it is desirable to retard tissue waste as much as possible.

[1] Ztchr. f. Klin. Med. xxiv. Hft. 3 and 4, 1894. [2] Therap. Monatshefte, 1887. [3] Dissertation. St. Petersburg, 1890. [4] La Terapia Moderna iv., 1890. [5] Wildermuth Neurolog. Centralbl, viii., 15, 1889; and Nacke Allgem. Ztchr. f. Psychiat, (47), 1890. [6] Peiser Fortschr. der Med., 1893.

